# Methyl 4,6-*O*-benzyl­idene-α-d-gluco­pyran­oside monohydrate

**DOI:** 10.1107/S2414314625009472

**Published:** 2025-11-06

**Authors:** Mari Grobbelaar, Eric Cyriel Hosten, Richard Betz

**Affiliations:** a Nelson Mandela University, Summerstrand Campus, Department of Chemistry, University Way, Summerstrand, PO Box 77000, Port Elizabeth, 6031, South Africa; Goethe-Universität Frankfurt, Germany

**Keywords:** gluco­pyran­ose, crystal structure, hydrogen bonds

## Abstract

The title compound, C_14_H_18_O_6_·H_2_O, is a partially protected derivative of d-gluco­pyran­ose. The asymmetric unit contains one sugar molecule and one water molecule of crystallization. Classical hydrogen bonds of the O—H⋯O type form a cooperative set and are observed next to a C—H⋯O(water) contact, connecting the entities of the asymmetric unit into a three-dimensional network.

## Abstract 2

The title compound is a hydrated, partially protected derivative of d-gluco­pyran­ose. Inter­molecular inter­actions connect the entities of the asymmetric unit to a three-dimensional network.

## Graphical abstract

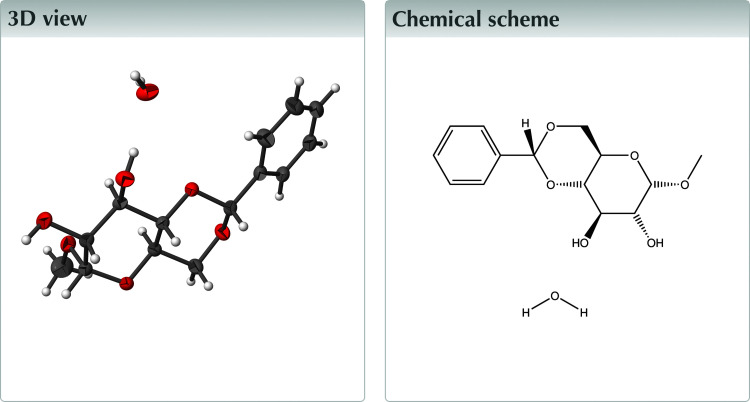

## Structure description

Carbohydrates are an important group of biomolecules and form part of the three macronutrients of the human diet. Natural members of this compound class of polyhy­droxy­carbonyls abound for derivatives with five and six carbon atoms, whose stereochemical diversity is enriched by the ability to form furan­oid and pyran­oid intra­molecular hemiacetal-type addition compounds. As they are the product of natural photosynthesis, they are debated as renewable and carbon-neutral feedstock materials for many industrial processes; however, precisely because of their stereochemical variability, exploiting their synthetic potential often requires a carefully crafted preparative strategy based on protection group chemistry (Lindhorst, 2003 ▸).

In connection with the synthesis of coordination compounds, limiting the number of potential donor sites on a polyfunctional carbohydrate is an important measure to ensure the formation of well-defined product species. In connection with a research project around the coordination behaviour of certain hexoses, partially protected derivatives of d-glucose were to be investigated with a specific focus on the *trans*-orientated hydroxyl groups on the six-membered ring. To this end, methyl-4,6-*O*-benzyl­idene-α-d-gluco­pyran­oside was synthesized and characterized in the solid state to allow for the comparison of metrical parameters in the free ligand and in coordination compounds. The structure of the title compound has been reported earlier (Tamaru *et al.*, 2001 ▸) but no three-dimensional coordinates were deposited. However, structural information is at hand for the anhydrous version of the title compound (Luboradzki *et al.*, 2000 ▸) as well as the β-anomer of the carbohydrate (Jessen *et al.*, 2001 ▸). The stereoisomeric altro­pyran­oside (Bozo & Vasella, 1992 ▸), allo­pyran­oside (Muddasani *et al.*, 1994 ▸) and ido­pyran­oside (Chu & Jeffrey, 1965 ▸; Liu *et al.*, 1993 ▸; Orban *et al.*, 2023 ▸) equivalents of the title compound have been the focus of diffraction studies on single crystals previously. The present study is a continuation of our inter­est in structural aspects of coordination compounds of carbohydrate derivatives (Betz & Klüfers, 2007*a* ▸, 2009 ▸; Betz *et al.*, 2007*a* ▸) as well as polyheterocyclic compounds (Muller *et al.*, 2021 ▸; Betz & Klüfers, 2007*b* ▸,*c* ▸,*d* ▸; Betz & Klüfers, 2008*a* ▸,*b* ▸; Betz *et al.*, 2007*b* ▸) and intends to close the gap of missing three-dimensional coordinates for the title compound.

**Figure 1:**
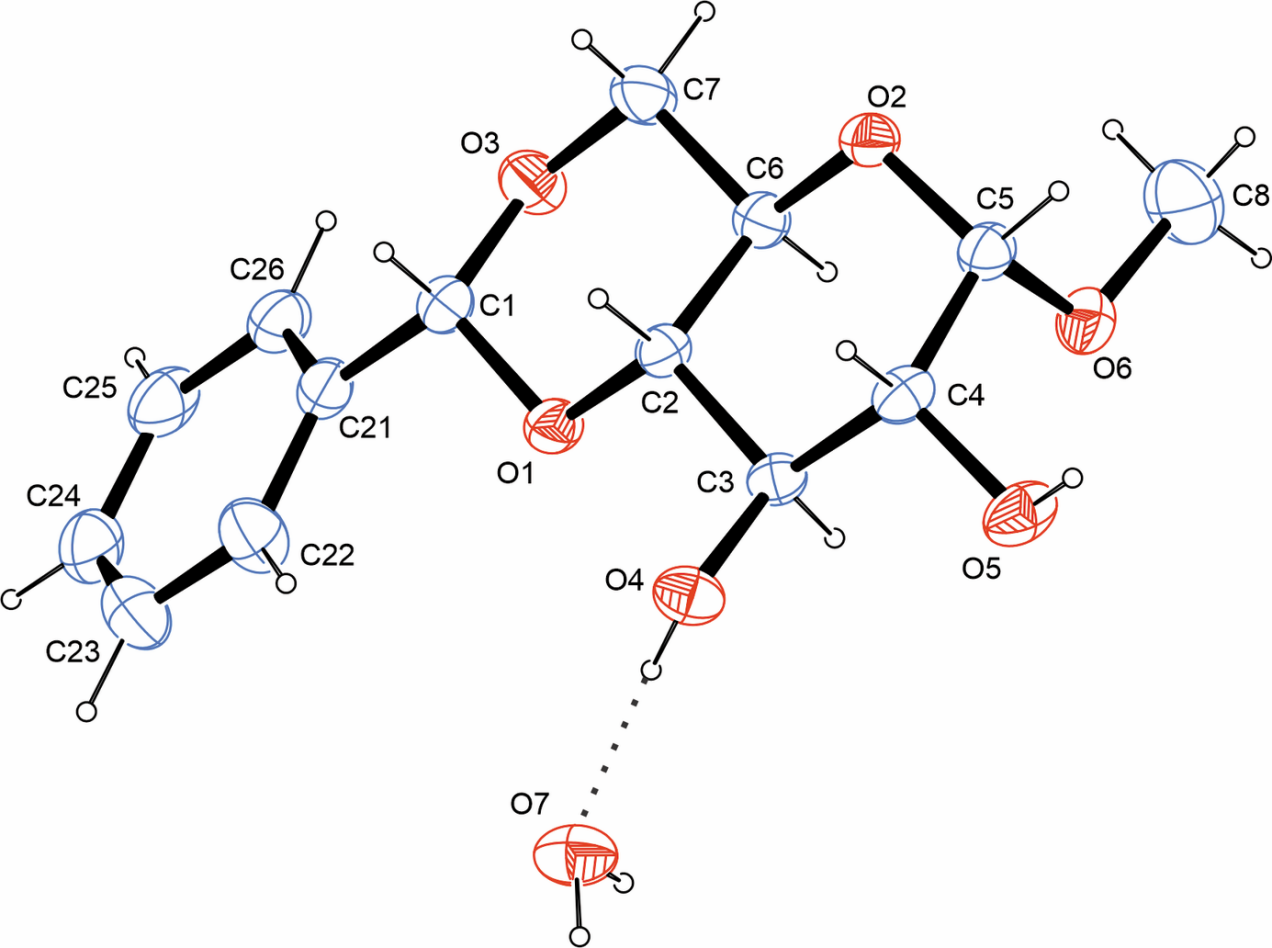
The mol­ecular structure of the title compound, with atom labels and anisotropic displacement ellipsoids (drawn at the 50% probability level).

The title compound (Fig. 1 ▸) is a twofold protected derivative of d-gluco­pyran­ose with the anomeric hydroxyl group converted into a meth­oxy group (O6–C8) and the hy­droxy­methyl and the adjacent ring-bound hydroxyl group capped by a benzyl­idene protection group. The two *trans*-orientated hydroxyl groups on the pyran­ose ring remain free. A mol­ecule of water is present in the asymmetric unit. Bond lengths and angles are in good agreement with values reported for comparable compounds whose metrical parameters have been elucidated by means of diffraction studies on single crystals and deposited with the Cambridge Structural Database (Groom *et al.*, 2016 ▸). While the meth­oxy group occupies an axial position, the phenyl group is found in an equatorial position. The two free hydroxyl groups adopt a staggered conformation with a O4—C3—C4—O5 torsion angle of 65.17 (16)°. A conformational analysis of the six-membered rings according to Cremer & Pople (1975 ▸) shows the pyran­oid ring to adopt a ^1^*C*_4_ (^O2^*C*_C3_) conformation while the six-membered ring established by the condensed benzyl­idene protection group is present in a ^4^*C*_1_ (^C7^*C*_O1_) conformation (Boeyens, 1978 ▸).

**Figure 2:**
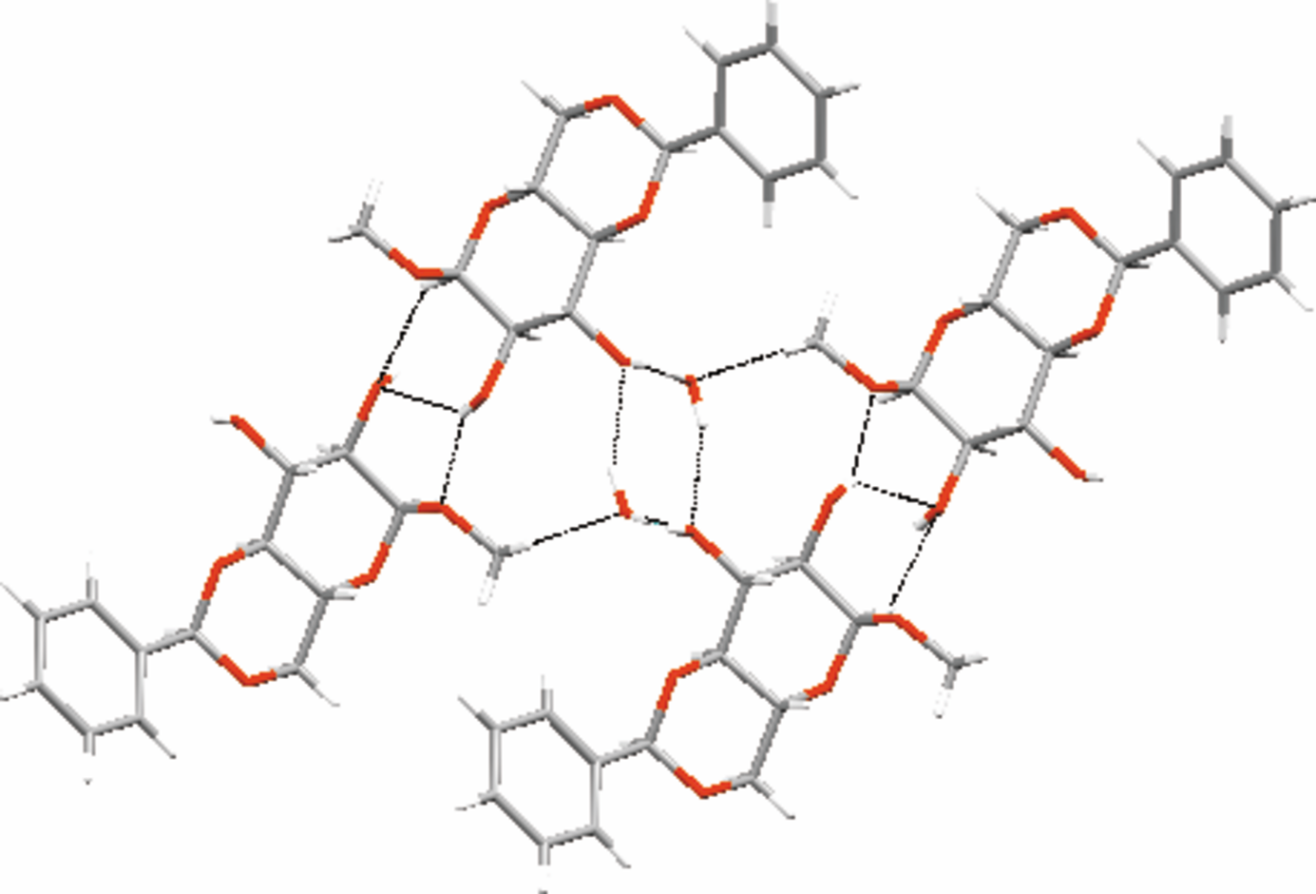
Selected inter­molecular contacts, viewed along [010].

In the crystal, classical hydrogen bonds of the O—H⋯O type are observed next to a C—H⋯O(water) contact (Table 1 ▸) whose range falls by more than 0.1 Å below the sum of van der Waals radii of the atoms participating in them. The hydroxyl group adjacent to the anomeric center establishes a hydrogen bond to the oxygen atom of the meth­oxy group as acceptor, while the second free hydroxyl group involves the oxygen atom of the free water mol­ecule as acceptor. The water mol­ecule exclusively forms hydrogen bonds to the oxygen atom of the second free hydroxyl group, thus giving rise to a cooperative set of hydrogen bonds. The C—H⋯O(water) contact is observed between one of the hydrogen atoms of the meth­oxy group as donor and solvent mol­ecule’s oxygen atom as acceptor. A second C—H⋯O contact between the hydrogen atom of the anomeric center’s methine group and the oxygen atom of the hydroxyl group adjacent to the anomeric center is listed for completeness but could be considered an artefact (or consequence) of the hydrogen bonds established by the neighbouring hydroxyl group resulting in distance shortening between the respective C—H and O motifs involved. In terms of graph-set analysis (Etter *et al.*, 1990 ▸; Bernstein *et al.*, 1995 ▸), the classical hydrogen bonds require a *DDDC^1^_1_(5)* descriptor on the unary level while the C—H⋯O contacts require a *DC^1^_1_(4)* descriptor on the same level with the finite pattern reserved for the water-based contact. Furthermore, one C—H⋯π contact is apparent in between the hydrogen atom of the benzyl­idene protection group and the aromatic system that connects the mol­ecules to chains along the crystallographic *b* axis. π-Stacking is not a stabilizing factor in the crystal structure of the title compound with the shortest distance in between two centers of gravity measured at 4.8475 (13) Å, which is in agreement with the length of the *b* axis of the unit cell (Fig. 2 ▸).

## Synthesis and crystallization

The compound was obtained following published standard procedures (Becker *et al.*, 2000 ▸; Lindhorst, 2003 ▸; Evans, 1972 ▸). Crystals suitable for the diffraction study were obtained upon recrystallization from boiling propan-2-ol containing water (alcohol:water approximately 95:5).

## Refinement

Crystal data, data collection and structure refinement details are summarized in Table 2 ▸.

## Supplementary Material

Crystal structure: contains datablock(s) I. DOI: 10.1107/S2414314625009472/bt4184sup1.cif

Structure factors: contains datablock(s) I. DOI: 10.1107/S2414314625009472/bt4184Isup2.hkl

CCDC reference: 2498654

Additional supporting information:  crystallographic information; 3D view; checkCIF report

## Figures and Tables

**Table 1:** Hydrogen-bond geometry (Å, °) *C*_g_(1) is the centroid of carbon atoms C21–C26.

*D*—H⋯*A*	*D*—H	H⋯*A*	*D*⋯*A*	*D*—H⋯*A*
O4—H4*A*⋯O7	0.84	1.86	2.6925 (19)	172
O5—H5*A*⋯O5^i^	0.84	2.48	3.1906 (18)	143
O5—H5*A*⋯O6^i^	0.84	2.12	2.8486 (18)	145
O7—H7*C*⋯O4^ii^	0.84 (1)	2.08 (2)	2.8758 (17)	159 (3)
O7—H7*D*⋯O4^iii^	0.83 (1)	2.02 (1)	2.8444 (19)	175 (3)
C5—H5⋯O5^i^	1.00	2.46	3.299 (2)	141
C8—H8*C*⋯O7^iv^	0.98	2.43	3.380 (3)	163
C1—H1⋯*C*_g_(1)^v^	1.00	2.56	3.516 (2)	161

Symmetry codes: (i) 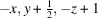; (ii) 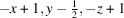; (iii) 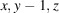; (iv) 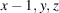; (v) 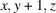.

**Table 2:** Experimental details

Crystal data	
Chemical formula	C_14_H_18_O_6_·H_2_O
*M*_r_	300.30
Crystal system, space group	Monoclinic, *P*2_1_
Temperature (K)	200
*a*, *b*, *c* (Å)	8.9794 (6), 4.8475 (3), 17.3824 (11)
β (°)	103.927 (2)
*V* (Å^3^)	734.37 (8)
*Z*	2
Radiation type	Mo *K*α
μ (mm^−1^)	0.11
Crystal size (mm)	0.50 × 0.13 × 0.07

Data collection	
Diffractometer	Bruker APEXII CCD
Absorption correction	Multi-scan (*SADABS*; Krause *et al.*, 2015 ▸)
*T*_min_, *T*_max_	0.705, 0.746
No. of measured, independent and observed [*I* > 2σ(*I*)] reflections	41341, 3652, 3442
*R*_int_	0.033
(sin θ/λ)_max_ (Å^−1^)	0.667

Refinement	
*R*[*F*^2^ > 2σ(*F*^2^)], *wR*(*F*^2^), *S*	0.030, 0.081, 1.06
No. of reflections	3652
No. of parameters	204
No. of restraints	4
H-atom treatment	H atoms treated by a mixture of independent and constrained refinement
Δρ_max_, Δρ_min_ (e Å^−3^)	0.25, −0.16
Absolute structure	Flack *x* determined using 1467 quotients [(*I*^+^)−(*I*^−^)]/[(*I*^+^)+(*I*^−^)] (Parsons *et al.*, 2013 ▸)
Absolute structure parameter	−0.17 (16)

Computer programs: *APEX2* and *SAINT* (Bruker, 2014 ▸), *SHELXS97* (Sheldrick 2008 ▸), *ORTEP-3 for Windows* (Farrugia, 2012 ▸), *Mercury* (Macrae *et al.*, 2020 ▸), *SHELXL2019/3* (Sheldrick, 2015 ▸) and *PLATON* (Spek, 2020 ▸).

## full crystallographic data

### Methyl 4,6-O-benzylidene-α-D-glucopyranoside monohydrate Crystal data

**Table:**

C_14_H_18_O_6_·H_2_O	*F*(000) = 320
*M**_r_* = 300.30	*D*_x_ = 1.358 Mg m^−^^3^
Monoclinic, *P*2_1_	Mo *K*α radiation, λ = 0.71073 Å
*a* = 8.9794 (6) Å	Cell parameters from 9923 reflections
*b* = 4.8475 (3) Å	θ = 2.4–28.3°
*c* = 17.3824 (11) Å	µ = 0.11 mm^−^^1^
β = 103.927 (2)°	*T* = 200 K
*V* = 734.37 (8) Å^3^	Rod, colourless
*Z* = 2	0.50 × 0.13 × 0.07 mm

### Methyl 4,6-O-benzylidene-α-D-glucopyranoside monohydrate Data collection

**Table:**

Bruker APEXII CCD diffractometer	3652 independent reflections
Radiation source: sealed tube	3442 reflections with *I* > 2σ(*I*)
Graphite monochromator	*R*_int_ = 0.033
φ and ω scans	θ_max_ = 28.3°, θ_min_ = 2.3°
Absorption correction: multi-scan (SADABS; Krause *et al.*, 2015)	*h* = −11→11
*T*_min_ = 0.705, *T*_max_ = 0.746	*k* = −6→6
41341 measured reflections	*l* = −23→23

### Methyl 4,6-O-benzylidene-α-D-glucopyranoside monohydrate Refinement

**Table:**

Refinement on *F*^2^	Hydrogen site location: mixed
Least-squares matrix: full	H atoms treated by a mixture of independent and constrained refinement
*R*[*F*^2^ > 2σ(*F*^2^)] = 0.030	*w* = 1/[σ^2^(*F*_o_^2^) + (0.0395*P*)^2^ + 0.1456*P*] where *P* = (*F*_o_^2^ + 2*F*_c_^2^)/3
*wR*(*F*^2^) = 0.081	(Δ/σ)_max_ < 0.001
*S* = 1.06	Δρ_max_ = 0.25 e Å^−^^3^
3652 reflections	Δρ_min_ = −0.16 e Å^−^^3^
204 parameters	Extinction correction: SHELXL-2019/2 (Sheldrick, 2015), Fc^*^=kFc[1+0.001xFc^2^λ^3^/sin(2θ)]^-1/4^
4 restraints	Extinction coefficient: 0.019 (5)
Primary atom site location: structure-invariant direct methods	Absolute structure: Flack *x* determined using 1467 quotients [(*I*^+^)-(*I*^-^)]/[(*I*^+^)+(*I*^-^)] (Parsons *et al.*, 2013)
Secondary atom site location: difference Fourier map	Absolute structure parameter: −0.17 (16)

### Methyl 4,6-O-benzylidene-α-D-glucopyranoside monohydrate Special details

**Table:**

Geometry. All esds (except the esd in the dihedral angle between two l.s. planes) are estimated using the full covariance matrix. The cell esds are taken into account individually in the estimation of esds in distances, angles and torsion angles; correlations between esds in cell parameters are only used when they are defined by crystal symmetry. An approximate (isotropic) treatment of cell esds is used for estimating esds involving l.s. planes.
Refinement. The carbon-bound H atoms were placed in calculated positions (C–H 0.98 Å for the methyl group, C–H 0.99 Å for the methylene group and C–H 1.00 Å for the methine groups) and were included in the refinement in the riding model approximation, with *U*(H) set to 1.2*U*_eq_(C).The H atoms of the methyl group were allowed to rotate with a fixed angle around the C–O bond to best fit the experimental electron density (HFIX 137 in the *SHELX* program suite (Sheldrick, 2015), with *U*(H) set to 1.5*U*_eq_(C).The H atoms of the hydroxyl groups were allowed to rotate with a fixed angle around the C–O bond to best fit the experimental electron density (HFIX 147 in the *SHELX* program suite (Sheldrick, 2015)), with *U*(H) set to 1.5*U*_eq_(O).The hydrogen atoms of the water molecule were located on a difference Fourier map and refined freely with the O—H bonds restrained to 0.84 (1) Å and the H···H distance restrained to 1.34 (2) Å.

### Methyl 4,6-O-benzylidene-α-D-glucopyranoside monohydrate Fractional atomic coordinates and isotropic or equivalent isotropic displacement parameters (Å^2^)

**Table:**

	*x*	*y*	*z*	*U*_iso_*/*U*_eq_	
O1	0.46554 (13)	0.5554 (2)	0.74863 (6)	0.0238 (3)	
O2	0.12794 (13)	0.9982 (3)	0.71868 (7)	0.0254 (3)	
O3	0.40114 (14)	0.6184 (3)	0.87039 (7)	0.0314 (3)	
O4	0.42598 (14)	0.7276 (3)	0.58470 (7)	0.0276 (3)	
H4A	0.471173	0.574777	0.588148	0.044 (7)*	
O5	0.11857 (16)	0.8527 (3)	0.50945 (7)	0.0326 (3)	
H5A	0.073092	0.989590	0.484881	0.043 (7)*	
O6	−0.02371 (13)	0.7058 (3)	0.62439 (7)	0.0297 (3)	
O7	0.57272 (16)	0.2455 (3)	0.58050 (8)	0.0348 (3)	
C1	0.52160 (18)	0.6135 (4)	0.83089 (9)	0.0244 (3)	
H1	0.574134	0.796858	0.837146	0.029*	
C2	0.36312 (17)	0.7704 (3)	0.71266 (9)	0.0206 (3)	
H2	0.418578	0.950973	0.720224	0.025*	
C3	0.30499 (17)	0.7132 (3)	0.62492 (9)	0.0206 (3)	
H3	0.257645	0.525372	0.617432	0.025*	
C4	0.18457 (19)	0.9274 (3)	0.58932 (9)	0.0233 (3)	
H4	0.236211	1.110364	0.589884	0.028*	
C5	0.06150 (19)	0.9515 (3)	0.63686 (10)	0.0244 (3)	
H5	−0.007931	1.109256	0.615502	0.029*	
C6	0.23033 (18)	0.7808 (3)	0.75229 (9)	0.0233 (3)	
H6	0.174321	0.600724	0.744613	0.028*	
C7	0.2933 (2)	0.8339 (4)	0.84008 (10)	0.0312 (4)	
H7A	0.344631	1.015908	0.848362	0.037*	
H7B	0.208970	0.832990	0.867783	0.037*	
C8	−0.1524 (2)	0.7112 (6)	0.65891 (16)	0.0531 (6)	
H8A	−0.213356	0.877348	0.641402	0.080*	
H8B	−0.116555	0.712809	0.716801	0.080*	
H8C	−0.215818	0.547268	0.642267	0.080*	
C21	0.63591 (19)	0.3933 (4)	0.86650 (10)	0.0259 (3)	
C22	0.7440 (2)	0.3128 (5)	0.82542 (12)	0.0357 (4)	
H22	0.740831	0.389879	0.774803	0.043*	
C23	0.8563 (2)	0.1213 (5)	0.85770 (13)	0.0411 (5)	
H23	0.931025	0.071158	0.829763	0.049*	
C24	0.8597 (2)	0.0029 (5)	0.93049 (12)	0.0392 (5)	
H24	0.936355	−0.128995	0.952543	0.047*	
C25	0.7510 (2)	0.0775 (4)	0.97097 (11)	0.0357 (4)	
H25	0.751721	−0.006604	1.020429	0.043*	
C26	0.6400 (2)	0.2755 (4)	0.93970 (10)	0.0300 (4)	
H26	0.567370	0.329480	0.968532	0.036*	
H7C	0.599 (3)	0.242 (7)	0.5371 (11)	0.063 (8)*	
H7D	0.527 (3)	0.099 (4)	0.5837 (15)	0.049 (7)*	

### Methyl 4,6-O-benzylidene-α-D-glucopyranoside monohydrate Atomic displacement parameters (Å^2^)

**Table:**

	*U*^11^	*U*^22^	*U*^33^	*U*^12^	*U*^13^	*U*^23^
O1	0.0261 (5)	0.0249 (6)	0.0194 (5)	0.0041 (5)	0.0033 (4)	−0.0010 (4)
O2	0.0268 (6)	0.0252 (6)	0.0228 (5)	0.0056 (5)	0.0032 (4)	−0.0036 (5)
O3	0.0303 (6)	0.0431 (8)	0.0214 (5)	0.0104 (6)	0.0075 (5)	0.0041 (5)
O4	0.0324 (6)	0.0276 (6)	0.0260 (6)	0.0030 (5)	0.0134 (5)	0.0026 (5)
O5	0.0455 (7)	0.0300 (7)	0.0183 (5)	0.0057 (6)	−0.0001 (5)	0.0007 (5)
O6	0.0252 (5)	0.0319 (7)	0.0309 (6)	−0.0053 (5)	0.0050 (4)	−0.0046 (5)
O7	0.0444 (7)	0.0294 (7)	0.0362 (7)	−0.0025 (6)	0.0207 (6)	−0.0032 (6)
C1	0.0241 (7)	0.0283 (8)	0.0195 (7)	0.0012 (7)	0.0030 (6)	−0.0016 (6)
C2	0.0228 (7)	0.0189 (7)	0.0196 (6)	0.0001 (6)	0.0039 (5)	−0.0010 (6)
C3	0.0248 (7)	0.0172 (7)	0.0207 (6)	−0.0014 (6)	0.0070 (5)	−0.0008 (6)
C4	0.0304 (8)	0.0176 (8)	0.0204 (7)	−0.0005 (6)	0.0033 (6)	0.0006 (6)
C5	0.0258 (8)	0.0226 (8)	0.0229 (7)	0.0023 (6)	0.0019 (6)	−0.0015 (6)
C6	0.0235 (7)	0.0245 (8)	0.0216 (7)	0.0021 (6)	0.0047 (6)	−0.0005 (6)
C7	0.0301 (8)	0.0410 (10)	0.0224 (7)	0.0110 (8)	0.0064 (6)	−0.0006 (7)
C8	0.0343 (10)	0.0628 (16)	0.0679 (15)	−0.0120 (11)	0.0235 (10)	−0.0125 (13)
C21	0.0246 (7)	0.0265 (8)	0.0242 (7)	−0.0005 (7)	0.0008 (6)	−0.0034 (6)
C22	0.0310 (9)	0.0427 (12)	0.0342 (9)	0.0064 (8)	0.0096 (7)	0.0068 (8)
C23	0.0303 (9)	0.0487 (12)	0.0438 (10)	0.0108 (9)	0.0080 (8)	−0.0016 (10)
C24	0.0350 (9)	0.0380 (11)	0.0372 (10)	0.0098 (9)	−0.0060 (8)	−0.0039 (9)
C25	0.0442 (10)	0.0344 (10)	0.0227 (8)	0.0058 (8)	−0.0034 (7)	−0.0005 (7)
C26	0.0322 (8)	0.0331 (10)	0.0219 (7)	0.0037 (7)	0.0011 (6)	−0.0023 (7)

### Methyl 4,6-O-benzylidene-α-D-glucopyranoside monohydrate Geometric parameters (Å, º)

**Table:**

O1—C1	1.4250 (18)	C4—C5	1.535 (2)
O1—C2	1.4305 (18)	C4—H4	1.0000
O2—C5	1.4218 (19)	C5—H5	1.0000
O2—C6	1.4280 (19)	C6—C7	1.516 (2)
O3—C1	1.4141 (19)	C6—H6	1.0000
O3—C7	1.436 (2)	C7—H7A	0.9900
O4—C3	1.4281 (17)	C7—H7B	0.9900
O4—H4A	0.8400	C8—H8A	0.9800
O5—C4	1.4191 (19)	C8—H8B	0.9800
O5—H5A	0.8400	C8—H8C	0.9800
O6—C5	1.404 (2)	C21—C26	1.387 (2)
O6—C8	1.425 (2)	C21—C22	1.392 (2)
O7—H7C	0.841 (12)	C22—C23	1.386 (3)
O7—H7D	0.832 (13)	C22—H22	0.9500
C1—C21	1.507 (2)	C23—C24	1.383 (3)
C1—H1	1.0000	C23—H23	0.9500
C2—C3	1.5140 (19)	C24—C25	1.382 (3)
C2—C6	1.515 (2)	C24—H24	0.9500
C2—H2	1.0000	C25—C26	1.396 (3)
C3—C4	1.519 (2)	C25—H25	0.9500
C3—H3	1.0000	C26—H26	0.9500
			
C1—O1—C2	109.23 (11)	O2—C6—C2	109.77 (12)
C5—O2—C6	111.43 (12)	O2—C6—C7	109.48 (13)
C1—O3—C7	111.18 (13)	C2—C6—C7	108.66 (12)
C3—O4—H4A	109.5	O2—C6—H6	109.6
C4—O5—H5A	109.5	C2—C6—H6	109.6
C5—O6—C8	112.74 (16)	C7—C6—H6	109.6
H7C—O7—H7D	107 (2)	O3—C7—C6	107.50 (14)
O3—C1—O1	111.33 (12)	O3—C7—H7A	110.2
O3—C1—C21	109.72 (13)	C6—C7—H7A	110.2
O1—C1—C21	108.27 (13)	O3—C7—H7B	110.2
O3—C1—H1	109.2	C6—C7—H7B	110.2
O1—C1—H1	109.2	H7A—C7—H7B	108.5
C21—C1—H1	109.2	O6—C8—H8A	109.5
O1—C2—C3	109.66 (12)	O6—C8—H8B	109.5
O1—C2—C6	108.61 (12)	H8A—C8—H8B	109.5
C3—C2—C6	110.24 (12)	O6—C8—H8C	109.5
O1—C2—H2	109.4	H8A—C8—H8C	109.5
C3—C2—H2	109.4	H8B—C8—H8C	109.5
C6—C2—H2	109.4	C26—C21—C22	119.16 (17)
O4—C3—C2	111.45 (12)	C26—C21—C1	122.25 (15)
O4—C3—C4	108.54 (12)	C22—C21—C1	118.55 (15)
C2—C3—C4	108.92 (12)	C23—C22—C21	120.60 (18)
O4—C3—H3	109.3	C23—C22—H22	119.7
C2—C3—H3	109.3	C21—C22—H22	119.7
C4—C3—H3	109.3	C24—C23—C22	120.16 (19)
O5—C4—C3	108.23 (13)	C24—C23—H23	119.9
O5—C4—C5	111.15 (13)	C22—C23—H23	119.9
C3—C4—C5	111.52 (12)	C25—C24—C23	119.63 (18)
O5—C4—H4	108.6	C25—C24—H24	120.2
C3—C4—H4	108.6	C23—C24—H24	120.2
C5—C4—H4	108.6	C24—C25—C26	120.48 (18)
O6—C5—O2	111.81 (13)	C24—C25—H25	119.8
O6—C5—C4	106.76 (13)	C26—C25—H25	119.8
O2—C5—C4	111.56 (13)	C21—C26—C25	119.94 (17)
O6—C5—H5	108.9	C21—C26—H26	120.0
O2—C5—H5	108.9	C25—C26—H26	120.0
C4—C5—H5	108.9		
			
C7—O3—C1—O1	−62.57 (18)	C5—O2—C6—C2	63.05 (16)
C7—O3—C1—C21	177.59 (14)	C5—O2—C6—C7	−177.76 (14)
C2—O1—C1—O3	62.49 (17)	O1—C2—C6—O2	178.67 (12)
C2—O1—C1—C21	−176.82 (13)	C3—C2—C6—O2	−61.17 (16)
C1—O1—C2—C3	179.03 (12)	O1—C2—C6—C7	58.97 (17)
C1—O1—C2—C6	−60.45 (15)	C3—C2—C6—C7	179.13 (14)
O1—C2—C3—O4	−65.88 (16)	C1—O3—C7—C6	59.08 (18)
C6—C2—C3—O4	174.60 (13)	O2—C6—C7—O3	−177.12 (13)
O1—C2—C3—C4	174.40 (12)	C2—C6—C7—O3	−57.25 (18)
C6—C2—C3—C4	54.88 (16)	O3—C1—C21—C26	−16.3 (2)
O4—C3—C4—O5	65.17 (16)	O1—C1—C21—C26	−138.00 (16)
C2—C3—C4—O5	−173.32 (13)	O3—C1—C21—C22	165.85 (16)
O4—C3—C4—C5	−172.26 (12)	O1—C1—C21—C22	44.2 (2)
C2—C3—C4—C5	−50.76 (16)	C26—C21—C22—C23	−1.2 (3)
C8—O6—C5—O2	63.6 (2)	C1—C21—C22—C23	176.72 (19)
C8—O6—C5—C4	−174.09 (16)	C21—C22—C23—C24	1.6 (3)
C6—O2—C5—O6	60.50 (16)	C22—C23—C24—C25	−0.3 (3)
C6—O2—C5—C4	−58.97 (17)	C23—C24—C25—C26	−1.4 (3)
O5—C4—C5—O6	51.55 (16)	C22—C21—C26—C25	−0.4 (3)
C3—C4—C5—O6	−69.32 (16)	C1—C21—C26—C25	−178.28 (17)
O5—C4—C5—O2	173.98 (13)	C24—C25—C26—C21	1.7 (3)
C3—C4—C5—O2	53.10 (17)		

### Methyl 4,6-O-benzylidene-α-D-glucopyranoside monohydrate Hydrogen-bond geometry (Å, º)

*C*_g_(1) is the centroid of carbon atoms C21–C26.

**Table:**

*D*—H···*A*	*D*—H	H···*A*	*D*···*A*	*D*—H···*A*
O4—H4*A*···O7	0.84	1.86	2.6925 (19)	172
O5—H5*A*···O5^i^	0.84	2.48	3.1906 (18)	143
O5—H5*A*···O6^i^	0.84	2.12	2.8486 (18)	145
O7—H7*C*···O4^ii^	0.84 (1)	2.08 (2)	2.8758 (17)	159 (3)
O7—H7*D*···O4^iii^	0.83 (1)	2.02 (1)	2.8444 (19)	175 (3)
C5—H5···O5^i^	1.00	2.46	3.299 (2)	141
C8—H8*C*···O7^iv^	0.98	2.43	3.380 (3)	163
C1—H1···*C*_g_(1)^v^	1.00	2.56	3.516 (2)	161

Symmetry codes: (i) −*x*, *y*+1/2, −*z*+1; (ii) −*x*+1, *y*−1/2, −*z*+1; (iii) *x*, *y*−1, *z*; (iv) *x*−1, *y*, *z*; (v) *x*, *y*+1, *z*.
